# Exploring the mechanisms of glycolytic genes involvement in pulmonary arterial hypertension through integrative bioinformatics analysis

**DOI:** 10.1111/jcmm.18447

**Published:** 2024-06-04

**Authors:** Yu‐xuan Lou, Er‐dan Shi, Rong Yang, Yang Yang

**Affiliations:** ^1^ Department of cardiology The First Affiliated Hospital with Nanjing Medical University Nanjing Jiangsu People's Republic of China; ^2^ Jiangsu Province Hospital of Chinese Medicine Affiliated Hospital of Nanjing University of Chinese Medicine Nanjing Jiangsu People's Republic of China; ^3^ Department of Rheumatology and Immunology Zhongda Hospital Affiliated to Southeast University Nanjing Jiangsu People's Republic of China

**Keywords:** bioinformatics, DEGs, glycolysis, machine learning, PAH

## Abstract

The purpose of this study was to identify the mechanisms underlying the involvement of glycolytic genes in pulmonary arterial hypertension (PAH). This study involved downloading 3 datasets from the GEO database at the National Center for Biotechnology Information. The datasets were processed to obtain expression matrices for analysis. Genes involved in glycolysis‐related pathways were obtained, and genes related to glycolysis were selected based on significant differences in expression. Gene Ontology functional annotation analysis, Kyoto Encyclopedia of Genes and Genomes pathway enrichment analysis, and GSEA enrichment analysis were performed on the DEGs. Combining LASSO regression with SVM‐RFE machine learning technology, a PAH risk prediction model based on glycolysis related gene expression was constructed, and CIBERSORTx technology was used to analyse the immune cell composition of PAH patients. Gene enrichment analysis revealed that the DEGs work synergistically across multiple biological pathways. A total of 6 key glycolysis‐related genes were selected using LASSO regression and SVM. A bar plot was constructed to evaluate the weights of the key genes and predict the risk of PAH. The clinical application value and predictive accuracy of the model were assessed. Immunological feature analysis revealed significant correlations between key glycolysis‐related genes and the abundances of different immune cell types. The glycolysis genes (ACSS2, ALAS2, ALDH3A1, ADOC3, NT5E, and TALDO1) identified in this study play important roles in the development of pulmonary arterial hypertension, providing new evidence for the involvement of glycolysis in PAH.

## 1 INTRODUCTION

Pulmonary arterial hypertension (PAH) is a condition characterized by a progressive increase in pulmonary vascular resistance, which ultimately leads to right heart failure and death. Epidemiological data on PAH from several international registry studies show an incidence of approximately 2.4 per million person‐years and a prevalence of approximately 15 per million in the adult population.^1^ The pathological changes of PAH include pulmonary artery media hypertrophy, concentric or eccentric intimal proliferation and fibrosis, adventitial thickening and fibrosis, perivascular inflammatory cell infiltration, and in situ thrombosis in the lumen. Changes in metabolism and bioenergetics are increasingly recognized as universal hallmarks of PAH in patients and in animal models of this disease.^2^

Glycolysis refers to the process in which glucose is broken down into pyruvate in the cytoplasm under anaerobic conditions; this process is a type of sugar metabolism. For a long time, the glycolytic pathway was shown to be related to the occurrence and development of cardiovascular diseases. For example, increased glycolysis is considered a marker of early metabolic remodelling in heart failure, and the glycolytic pathway is also the main source of energy for myocardial cells during ischemia–reperfusion.^3^ In addition, there is a relationship between glycolysis and the occurrence and development of PAH,^4^, ^5^ studies have found that tumour cells preferentially use glycolysis to promote their rapid growth, even under aerobic conditions, which is the Warburg effect. In recent years, more and more evidence shows that Warburg effect also occurs in non cancerous diseases such as PAH.^6^ Pulmonary vascular cells in PAH have a glycolytic phenotype, characterized by increased glycolysis and inhibition of glucose oxidation. Studies by Wang J and others have suggested that key glycolytic enzymes are an important part of the Warburg effect, and also play a crucial role in the pathogenesis of PAH.^7^

In recent years, with the development of bioinformation technology and machine learning theory systems, bioinformatics analysis methods and machine learning algorithms have been widely used to reveal the occurrence and development of diseases.^8^, ^9^, ^10^ In this study, we explored the mechanism by which glycolytic genes is involved in PAH via bioinformatics analysis and machine learning algorithms to provide a theoretical basis for identifying new therapeutic targets for PAH.

## 2 INFORMATION AND METHODOLOGY

### 2.1 Data source

The GSE113439,^11^ GSE53408,^12^ and GSE117261^13^ datasets were downloaded from the Gene Expression Omnibus (GEO) database (https://www.ncbi.nlm.nih.gov/geo/) of the National Center for Biotechnology Information (NCBI) in the U.S(Table 1). The GSE113439 and GSE117261 datasets were used as training sets, and the GSE53408 dataset was used as a validation set. The sequencing platforms used for these datasets were all from GPL6244 [HuGene‐1_0‐st] Affymetrix Human Gene 1.0 ST Array [transcript (gene) version]. The GSE113439 dataset was extracted and divided into 11 patients in the healthy control group (normal) and 15 patients in the PAH group.^11^ The GSE117261 dataset was extracted to obtain 25 cases from the control group (normal) and 58 cases from the PAH group.^13^ The GSE53408 dataset was extracted to obtain data from 11 patients in the control group (normal) and 12 patients in the PAH group; these patients composed the validation set.^12^ Subsequently, the GSE113439 and GSE117261 datasets were combined, and the datasets were processed through the ComBat function of the sva package^14^ for batch removal, standardization, and annotation of probes. The limma (3.58.1) package^15^ was used to correct the matrix to obtain the expression matrix for analysis. The genes involved in glycolysis‐related pathways were obtained from the Molecular Signatures Database (MSigDB) (https://www.gsea‐msigdb.org/gsea/msigdb/).^16^

**TABLE 1:** Overview of gene expression datasets for pulmonary arterial hypertension (PAH) lung tissue.

GEO accession	GPL platform	Sequencing type	Species	Tissue source	Group information
GSE113439	GPL6244	Transcriptomics	Human	Lung	11 Normal cases, 15 PAH cases
GSE117261	GPL6244	Transcriptomics	Human	Lung	25 Normal cases, 58 PAH cases
GSE53408	GPL6244	Transcriptomics	Human	Lung	11 Normal cases, 12 PAH cases

### 2.2 Research methodology

#### 2.2.1 Screening of differentially expressed PAH genes and PAH‐related gene modules

Based on the gene expression levels of the control group and the PAH group, the genes with significantly expressed genes (DEGs) in the control group and the PAH group were screened, and the genes with logFC >0.585 and *p* <0.05 were considered to be the genes associated with upregulated expression (up_ regulated_genes); the genes with logFC <−0.585 and *p* <0.05 were considered to be the genes associated with downregulated expression. The selected thresholds were |log2(FC)| >0.585 and *p* <0.05, and the data are displayed in a volcano plot. The expression profiles of glycolysis‐related genes were further extracted, and the genes related to glycolysis were differentially expressed between the PAH group and the control group with thresholds of |log2(FC)| >0.3 and *p* <0.05. The pheatmap package was subsequently used to evaluate differences in the expression of the genes between the control group and the PAH group.

#### 2.2.2 GO functional enrichment, Kyoto encyclopedia of genes and genomes (KEGG) pathway enrichment and GSEA enrichment analysis

The ‘clusterProfiler’ package in R software^17^ was used to perform GO functional annotation analysis and KEGG pathway enrichment analysis of the DEGs related to glycolysis. Obtain the ‘c2. cp. v7.5.1. symbols. The gmt gene set was obtained from the MSigDB (v7.5.1),^18^ and gene set enrichment analysis (GSEA) was performed on the DEGs.

#### 2.2.3 Construction of disease risk models

LASSO regression and support vector machines (SVMs) screening of key genes: The ‘glmnet’ package was used to complete the LASSO regression algorithm to determine whether the same parameters were significantly different between the group and the control group.^19^ The SVM is a supervised machine learning technique widely used in classification and regression analysis. We used SVM recursive feature elimination (SVM‐RFE) to screen for key genes.^20^ The results were analysed using the ‘Vennpackage in R software to determine the intersection of key genes, and common DEGs were obtained. Calibration curve analysis was carried out by the calibration function to verify the prediction accuracy of the model, and decision curve analysis (DCA) was performed through thermda’ package to evaluate the clinical application value of the model under different thresholds.^21^

#### 2.2.4 Immune cell infiltration analysis of PAHs

CIBERSORTx is based on the principle of linear support vector regression to deconvolute the transcriptome expression matrix, thereby estimating the composition and abundance of immune cells in a mixture of cells.^22^ The gene expression matrix data were uploaded to CIBERSORTx. The LM22 trait gene matrix was bound, the output of the *p* < 0.05 sample was filtered, and the immune cell infiltration matrix was obtained. A histogram was created using the R software ‘ggplot2’ package to show the distribution of 22 immune cell infiltrates in each sample. Subsequently, the Spearman correlation coefficient method was used to calculate the correlation between the infiltration of immune cells and DEGs, and the correlation heatmap was generated. By plotting boxplots to further observe the differences in immune cell infiltration between the different groups, the immune infiltration characteristics associated with the different PAHs could be further identified.

#### 2.2.5 mRNA–miRNA, mRNA‐TF and mRNA‐drug network construction and protein structure display of the hub genes

The miRNAs associated with key genes were obtained from the miRwalk (http://mirwalk.umm.uni‐heidelberg.de/)^23^ database, and the mRNA–miRNA regulatory network was constructed. The TRRUST (version 2) (https://www.grnpedia.org/trrust/) database was used to identify transcription factors that bind to the hub genes.^24^ The relationships between transcription factor targets and DEGs were retrieved, and an interaction network between key genes and TFs was constructed. The Drug–Gene Interaction Database (DGIdb) version 3.0.2 (https://www.dgidb.org) was used to predict potential drugs or small molecule compounds that interact with hub genes, and the mRNA–miRNA regulatory network was visualized using Cytoscape softwareKey gene–TF interaction networks and drug–gene interaction networks. The spatial structure of the core proteins of the key genes was obtained and displayed through the Molecular Modelling Database (MMDB).^25^

#### 2.2.6 Statistical analysis

All the data calculations and statistical analyses were performed with the use of R programming (https://www.r‐project.org/, version 4.1.2). For comparisons of continuous variables between two groups, the statistical significance of normally distributed variables was estimated with the use of an independent Student's t test, and differences between variables that were not normally distributed were analysed with the use of the Mann–Whitney *U* test (i.e. the Wilcoxon rank‐sum test). All the statistical *p* values were two‐sided, and a *p* value of less than 0.05 was used to indicate statistical significance.

## 3 OUTCOME

### 3.1 Screening of differentially expressed genes

**FIGURE 1:**
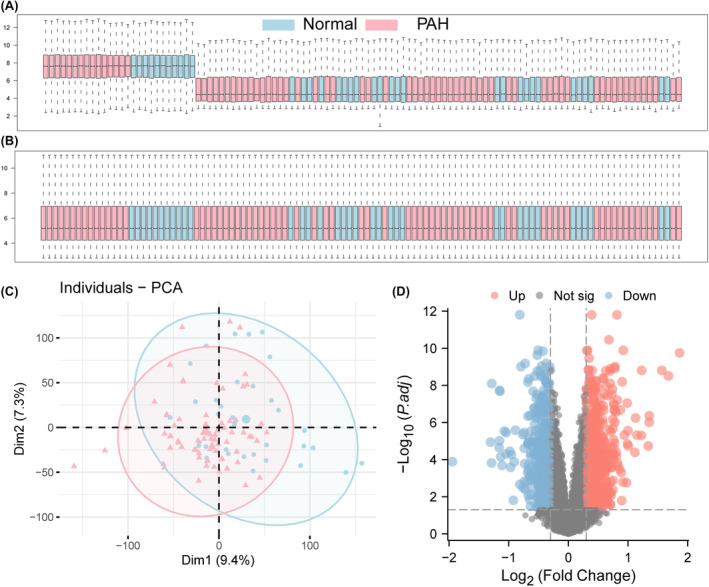
Analysis of differentially expressed genes in the dataset. (A) Distribution of expression profiles between samples from the two data sets before correction. (B) the distribution of expression profiles between the two data sets after correction, (C) Principal component analysis diagram between the disease group and the control group, where the pink points are the distribution of samples in the PAH group, and the blue points are the distribution of samples in the control group. (D) Volcano plot based on differential gene analysis, the abscisce is log2FoldChange, and the ordinate is‐log10 (p.ad). The red node represents the up‐regulated differentially expressed genes, the blue node represents the down‐regulated differentially expressed genes, and the black node represents the genes that are not significantly differentially expressed.

**FIGURE 2:**
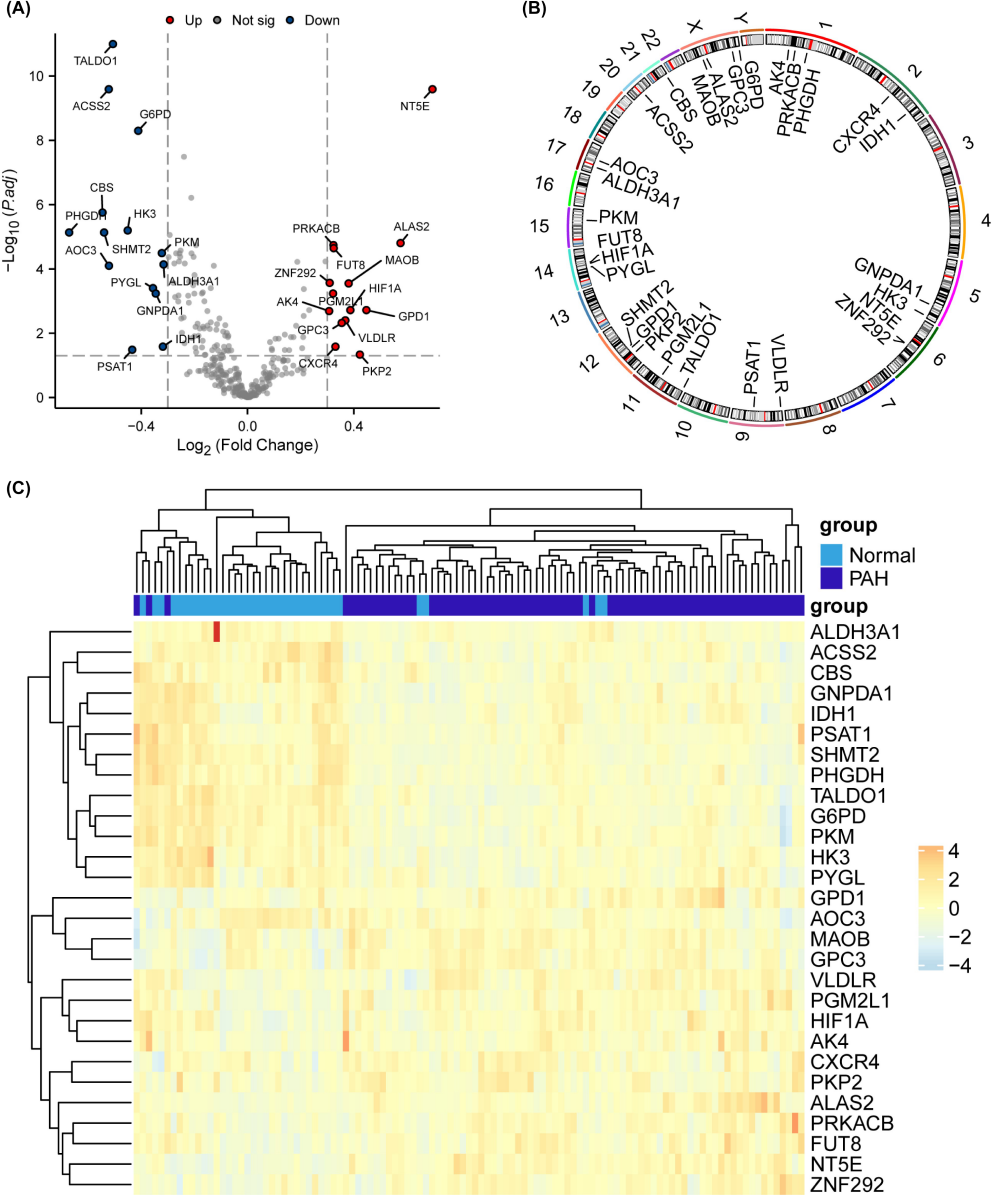
Differentially expressed genes related to glycolysis between PAH and controls. (A) Volcano plot of differential expression analysis of glycolysis‐related genes, with log2FoldChange on the abscisce and ‐log10 (P.adj) on the ordinate. Red nodes represent up‐regulated differentially expressed genes, blue nodes represent down‐regulated differentially expressed genes, and black nodes represent genes that are not significantly differentially expressed. (B) Chromosomal location distribution of differentially expressed glycolysis‐related genes. (C) Heat map analysis of differentially expressed genes related to glycolysis between PAH group and control group.

**FIGURE 3:**
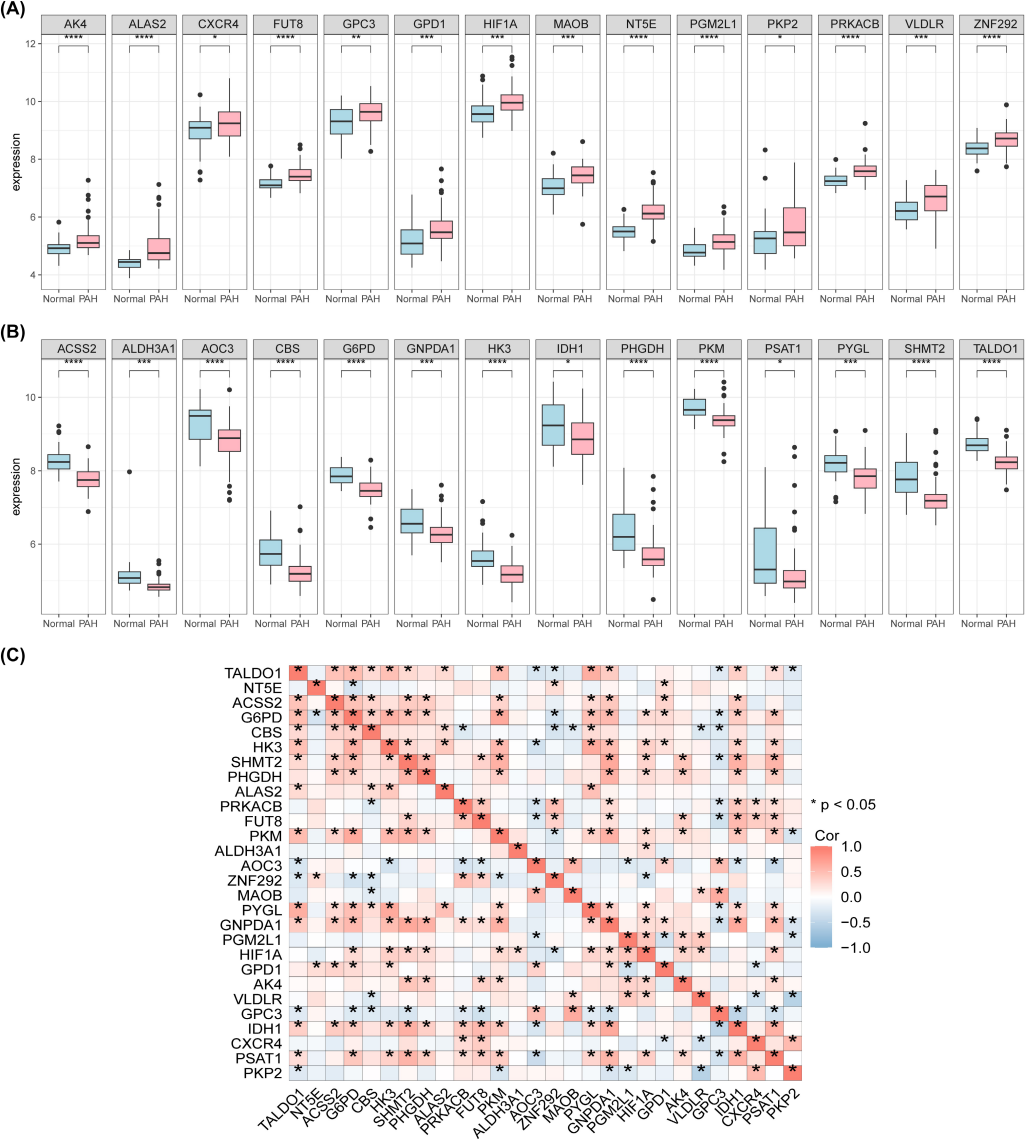
Differentially expressed genes related to glycolysis between PAH and controls. (A) Expression box plot of differentially expressed glycolysis‐related genes between PAH and control, where pink represents PAH group and blue represents control group. (B) Boxplot of differentially expressed glycolysis‐related genes between PAH and control group, pink represents PAH group and blue represents control group. C. Correlation analysis between differentially expressed glycolytic genes, the colour of each small square represents the correlation, the red colour represents the stronger positive correlation, the blue colour represents the stronger negative correlation. Asterisks in the small squares indicate the significance of the statistical difference. (* denotes *p* ≤ 0.05, ** denotes *p* ≤ 0.01, and *** denotes *p* ≤ 0.001).

After processing the dataset, 36 healthy control group samples and 73 PAH samples were obtained, and the distributions of the data were plotted before and after normalization (Figure 1A,B). A total of 353 glycolysis‐related genes were obtained from the MSigDB (https://www.gsea‐msigdb.org/gsea/msigdb/). PCA of the samples from the two groups revealed differences in the distribution of 169 related genes between the two groups (Figure 1C), and the differentially expressed genes were demonstrated by volcano plots, among which 109 were highly expressed (logFC was positive) and 60 were weakly expressed (logFC was negative). Through differential analysis of the expression matrix of glycolysis‐related genes, a total of 28 glycolysis‐related DEGs with significant differential expression between the PAH group and the normal group were screened (Figure 2A), and the positions of 28 genes on the chromosome are shown in Figure 2B. A heatmap (Figure 2C) and box plot (Figure 3A,B) were generated to show the differences in the expression of glycolysis genes between the PAH group and the normal group. The correlation between glycolytic genes is shown in Figure 3C.

### 3.2 GO functional enrichment, KEGG pathway enrichment and GSEA enrichment analysis of differentially expressed genes

**FIGURE 4:**
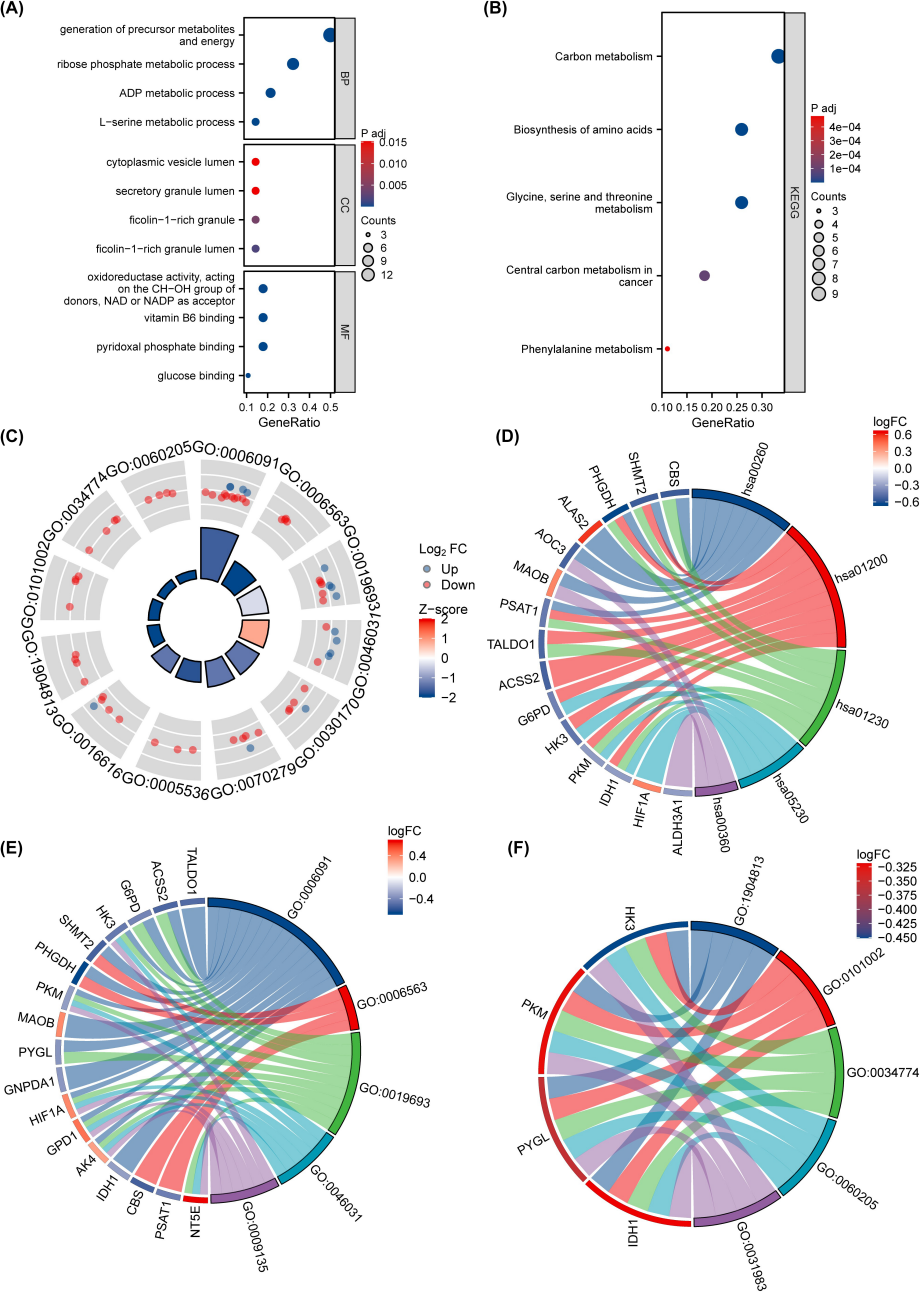
GO and KEGG enrichment analysis. (A) Dot plot of GO enrichment analysis of PAH‐related glycolytic genes, with GeneRatio on the abscissa and GO terms on the ordinate. (B). KEGG enrichment Pathway map of PAH‐related glycolytic genes, the abscissa is gene ratio, the ordinate is Pathway name, the node size represents the number of genes enriched in the pathway, and the node colour represents *p*value. (C) The visualization results of GO enrichment analysis circle diagram of PAH‐related glycolytic genes: the outer circle is GO terms, the red dots represent up‐regulated genes, the blue dots represent down‐regulated genes, the quadrate colour represents the zscore of GO terms, and the blue indicates that z‐score is negative, which means that the corresponding GO terms are more likely to be inhibited. Red indicates that z‐score is positive, which is more likely to be activated in the corresponding GO terms. (D) KEGG function enrichment chord diagram, node colour indicates gene expression level, red indicates up‐regulated genes, blue indicates down‐regulated genes. (E, F) are BP and CC functional enrichment chordplots, respectively, node colours indicate gene expression levels, red indicates up‐regulated genes, and blue indicates down‐regulated genes. (biological process, BP), (molecular function, MF), (cellular component, CC).

**FIGURE 5:**
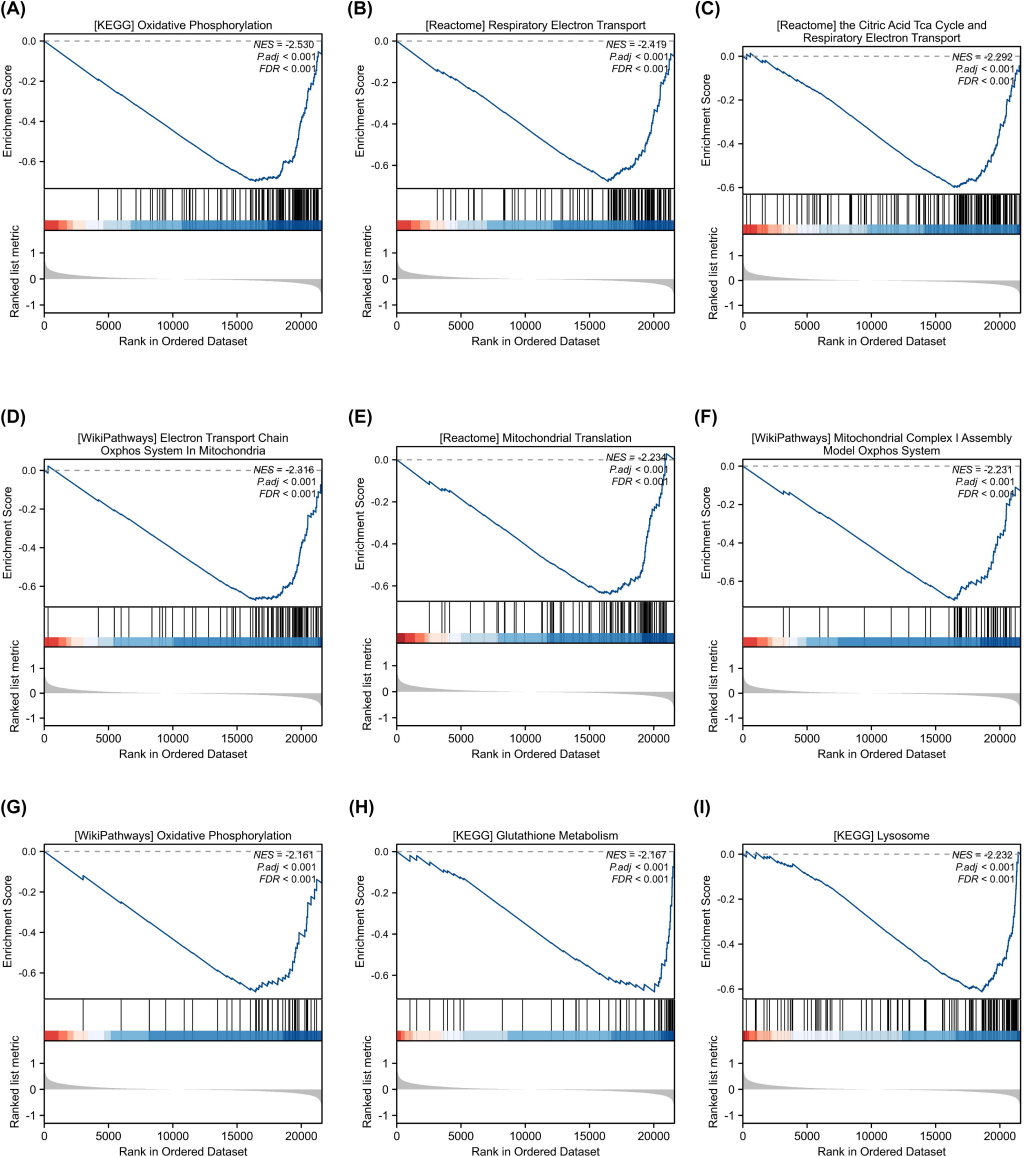
Gene set enrichment analysis (GSEA) method was used to rank the effects of different biological pathways. (A) GSEA results of KEGG oxidative phosphorylation pathways. (B) GSEA results of Reactome respiratory chain electron transport chain. (C) GSEA results of the Reactome tricarboxylic acid cycle and the electron transport chain of the respiratory chain. (D) GSEA results for the mitochondrial electron transport system in WikiPathways. (E) GSEA results for Reactome mitochondrial translation. (F) GSEA results for the OxPhos system of the WikiPathways mitochondrial complex assembly model. (G) GSEA results for the WikiPathways oxidative phosphorylation pathway. (H) GSEA results for KEGG glutathione metabolism. (I) GSEA results for KEGG lysosomes. In each figure, the blue line represents the change trend of enrichment scores, the vertical black line represents the position of each gene in the ordered dataset, while the horizontal red and blue bars show the distribution of gene sets in the rankings, where the red bar represents the positive contribution of enrichment scores and the blue bar represents the negative contribution. Enrichment score (ES), Normalized Enrichment Score (NES), False Discovery Rate (FDR). GSEA (Gene Set Enrichment Analysis).

GO analysis of differentially expressed glycolytic genes in PAH is shown in Table 2. GO analysis revealed enrichment in biological processes such as L‐serine metabolic process, ADP metabolic process, ribose phosphate metabolic process, generation of precursor metabolites and energy (Figure 4A, C, E), and ficolin‐1‐rich granule lumen, ficolin‐1‐rich granule, secretory granule lumen, cytoplasmic vesicle lumen, and other cellular fractions (Figure 4A, C, F); glucose binding; pyridoxal phosphate binding; vitamin B6 binding; and oxidoreductase activity, acting on the CH‐OH group of donors, NAD or NADP as acceptor (Figure 4A, C). KEGG pathway analysis of differentially expressed glycolytic genes in PAH is shown in Table 3. The results of KEGG pathway enrichment analysis showed that these genes were enriched in phenylalanine metabolism, central carbon metabolism in cancer, glycine, serine and threonine metabolism, biosynthesis of amino acids, carbon metabolism and other biological pathways (Figure 4B, D). GSEA in PAH is shown in Table 4. GSEA revealed the enrichment of the genes in the KEGG oxidative phosphorylation pathway (NES = −2.537, FDR <0.001) (Figure 5A) and the Reactome respiratory chain electron transport chain (NES = −2.079, FDR <0.001) (Figure 5B). The Reactome tricarboxylic acid cycle included the respiratory chain electron transport chain (NES = −2.492, FDR <0.001) (Figure 5C), the WikiPathways mitochondrial electron transport system (NES = −2.316, FDR <0.001) (Figure 5D), and the Reactome mitochondrial translation pathway (NES = −2.036)., (FDR <0.001) (Figure 5E); the WikiPathways mitochondrial complex assembly model OxPhos system (NES = −2.231, FDR <0.001) (Figure 5F); and the WikiPathways oxidative phosphorylation pathway (NES = −2.161, FDR <). 0.001) (Figure 5G), KEGG glutathione metabolism (NES = −2.167, FDR <0.001) (Figure 5H), and KEGG lysosomes (NES = −2.232, FDR <0.001) (Figure 5I) all showed negative enrichment scores. These results suggest that multiple glycolysis genes are involved in the occurrence and development of PAH.

**TABLE 2:** Gene ontology analysis of differentially expressed glycolysis genes in PAH.

Ontology	ID	Description	Gene ratio	Bg ratio	*p* value	*p*. adjust
BP	GO:0006091	generation of precursor metabolites and energy	14/28	494/18800	1.79 e‐15	1.88 e‐12
BP	GO:0006563	L‐serine metabolic process	4/28	12/18800	1.93 e‐09	9.25 e‐07
BP	GO:0019693	ribose phosphate metabolic process	9/28	394/18800	3.46 e‐09	9.25 e‐07
BP	GO:0046031	ADP metabolic process	6/28	90/18800	3.52 e‐09	9.25 e‐07
CC	GO:1904813	ficolin‐1‐rich granule lumen	4/28	124/19594	2.78 e‐05	0.0019
CC	GO:0101002	ficolin‐1‐rich granule	4/28	185/19594	0.0001	0.0044
CC	GO:0034774	secretory granule lumen	4/28	322/19594	0.0011	0.0152
CC	GO:0060205	cytoplasmic vesicle lumen	4/28	325/19594	0.0011	0.0152
MF	GO:0030170	pyridoxal phosphate binding	5/28	55/18410	1.84 e‐08	1.38 e‐06
MF	GO:0070279	vitamin B6 binding	5/28	56/18410	2.02 e‐08	1.38 e‐06
MF	GO:0005536	glucose binding	3/28	11/18410	5.16 e‐07	2.35 e‐05
MF	GO:0016616	oxidoreductase activity, acting on the CH‐OH group of donors, NAD or NADP as acceptor	5/28	128/18410	1.3 e‐06	4.45 e‐05

Abbreviations: BP, biological process; CC, cellular component; MF, molecular function.

**TABLE 3:** KEGG pathway analysis of differentially expressed glycolysis Genes in PAH.

Ontology	ID	Description	GeneRatio	BgRatio	*p* value	*p*. adjust
KEGG	hsa00260	Glycine, serine and threonine metabolism	7/27	40/8164	3.22 e‐11	4.1 e‐09
KEGG	hsa01200	Carbon metabolism	9/27	115/8164	6.03 e‐11	4.1 e‐09
KEGG	hsa01230	Biosynthesis of amino acids	7/27	75/8164	3.18 e‐09	1.44 e‐07
KEGG	hsa05230	Central carbon metabolism in cancer	5/27	70/8164	2.8 e‐06	9.51 e‐05
KEGG	hsa00360	Phenylalanine metabolism	3/27	16/8164	1.76 e‐05	0.0005

Abbreviations: KEGG, Kyoto encyclopedia of genes and genomes.

**TABLE 4:** Gene set enrichment analysis (GSEA) in PAH.

Description	NES	*p*value	*p*.adjust	*q* value
KEGG_OXIDATIVE_PHOSPHORYLATION	2.530214097	1E‐10	3.57143 e‐08	3.06917 e‐08
REACTOME_RESPIRATORY_ELECTRON_TRANSPORT	2.418664381	1E‐10	3.57143 e‐08	3.06917 e‐08
REACTOME_THE_CITRIC_ACID_TCA_CYCLE_AND_RESPIRATORY_ELECTRON_TRANSPORT	2.291825812	1E‐10	3.57143 e‐08	3.06917 e‐08
WP_ELECTRON_TRANSPORT_CHAIN_OXPHOS_SYSTEM_IN_MITOCHONDRIA	2.315997075	3.15937 e‐10	9.87304 e‐08	8.48458 e‐08
KEGG_LYSOSOME	2.23243756	1.08521 e‐09	2.71302 e‐07	2.33148 e‐07
REACTOME_MITOCHONDRIAL_TRANSLATION	2.234024591	4.02274 e‐09	9.14259 e‐07	7.85685 e‐07
WP_MITOCHONDRIAL_COMPLEX_I_ASSEMBLY_MODEL_OXPHOS_SYSTEM	2.231066044	1.9337 e‐07	2.41713 e‐05	2.0772 e‐05
WP_OXIDATIVE_PHOSPHORYLATION	2.161370454	1.89509 e‐06	0.000169205	0.000145409
KEGG_GLUTATHIONE_METABOLISM	2.166893714	2.54465 e‐06	0.000212054	0.000182233

### 3.3 Construction of PAH risk model

**FIGURE 6:**
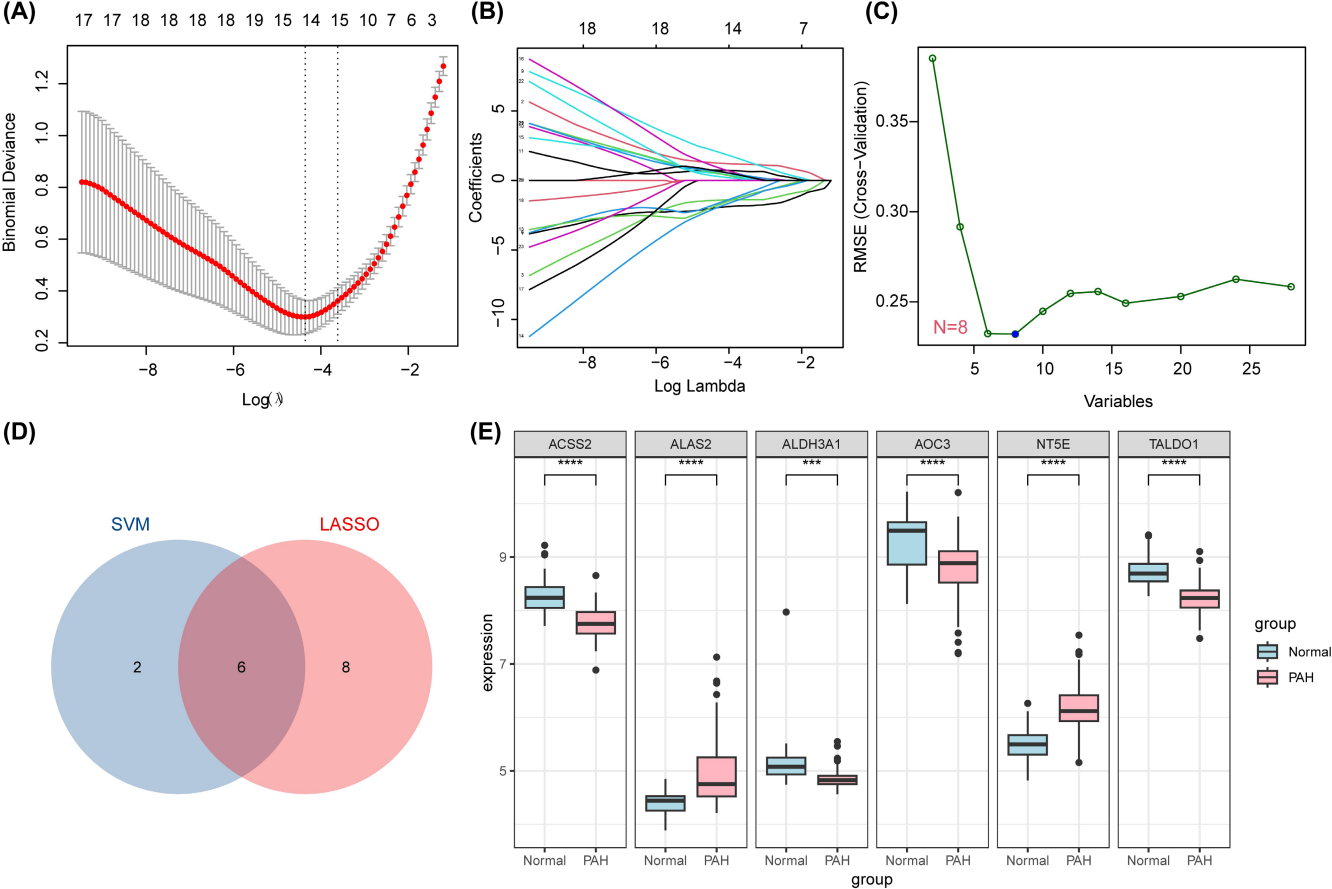
Identification and analysis of PAH biomarkers based on machine learning. (A) Plot of binomial distribution bias versus log (λ) of LASSO regression. The red dots in the plot indicate the binomial deviation for each λ value, and the grey area represents the range of standard errors. The dotted line represents the optimal λ value selected, where the corresponding binomial deviation is minimized, which is a compromise between the model complexity and the prediction accuracy. (B) Plot of coefficient paths of LASSO regression at different λ values. The coloured lines represent how the coefficients of different variables change as λ decreases (i.e. the complexity of the model increases). As λ decreases, more coefficients become nonzero, and the value of the coefficient gradually increases. (C) Plot of the influence of the number of variables in the SVM‐RFE method on the prediction performance of the model. The vertical axis represents the value of the root mean square error cross validation (RMSE CV), and the horizontal axis represents the number of variables selected by the recursive feature elimination method. When the number of variables is 8, the model achieves the lowest RMSE, indicating that this is the best combination of variables to achieve the optimal prediction performance. (D) Venn plot of the selected variables of SVM‐RFE versus LASSO model. Blue and red circles represent the variables selected by SVM and LASSO, respectively, and the cross section represents the number of variables selected by both methods. (E) Box plot of different gene expression between normal and PAH groups. Box plots show differences in expression levels of six genes (ACSS2, ALAS2, ALDH3A1, ADOC3, NT5E, TALDO1) at the intersection between the two groups, with black dots representing outliers. The upper and lower boundaries of the box plot indicate the first and third quartiles, and the horizontal line indicates the median. **** indicates a *p* value <0.0001, indicating a significant difference between the two groups. (SVM), (Least Absolute Shrinkage and Selection Operator, LASSO).

The DEGs in the dataset were analysed by the LASSO regression algorithm, and 14 key genes were screened out (Figure 6A,B). The DEGs in the dataset were analysed using the SVM machine learning algorithm, and 8 key genes were screened (Figure 6C). A Venn plot was constructed to reveal the numbers of key genes coselected by both methods, namely, ACSS2, ALAS2, ALDH3A1, ADOC3, NT5E, and TALDO1 (Figure 6D). Box plots revealed significant differences in the expression levels of six key glycolysis genes between the control and PAH groups (Figure 6E).

**FIGURE 7:**
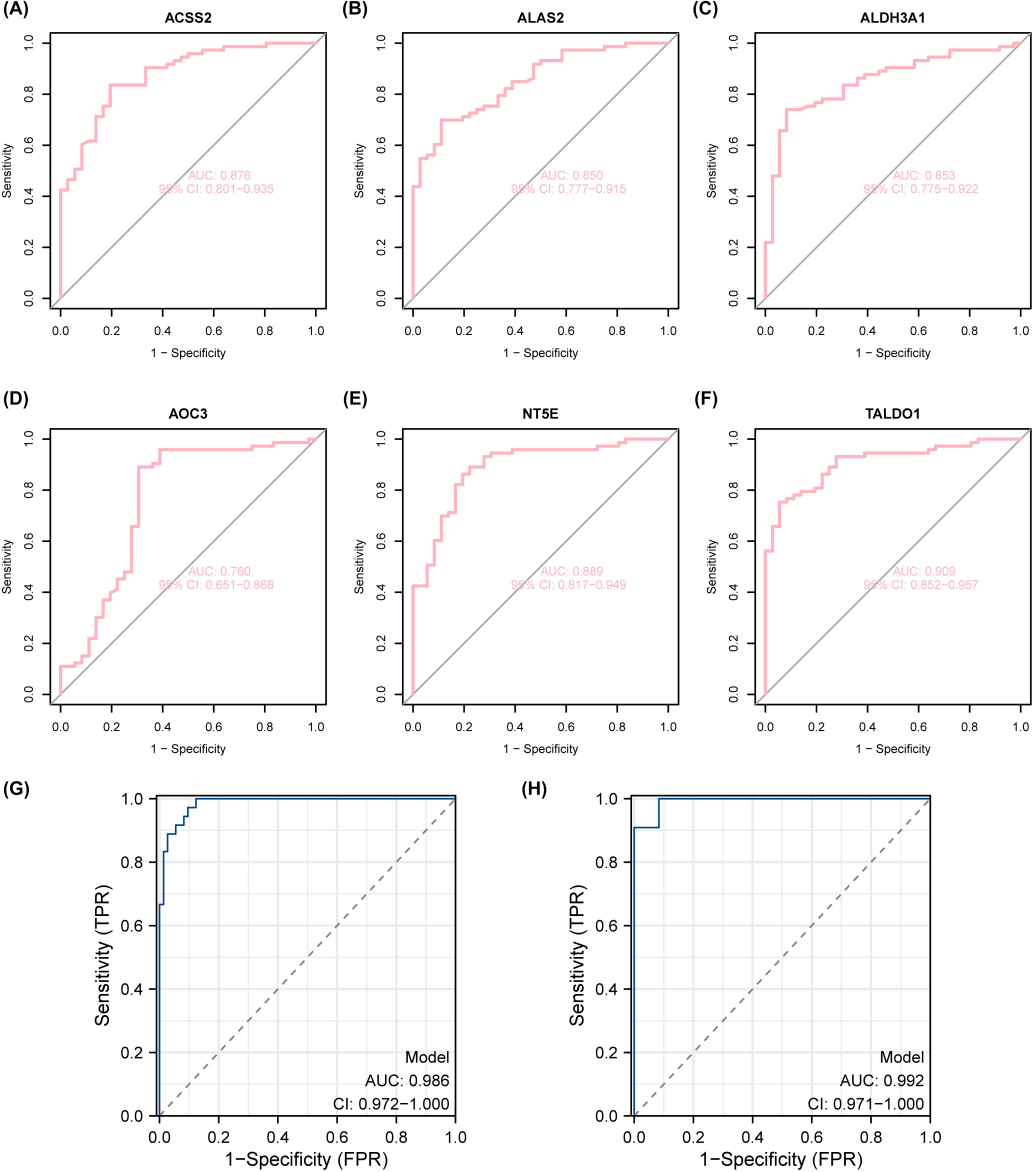
Evaluation of machine learning‐assisted biomarkers in the diagnosis of PAH. (A–F) Each panel presents the ROC curves of individual biomarkers for PAH. The area under the curve (AUC) and its 95% confidence interval (CI) were: (A) ACSS2:0.876 (CI: 0.801–0.935); (B) ALAS2:0.850 (CI: 0.777–0.915); (C) ALDH3A1:0.853 (CI: 0.775–0.922); (D) AOC3:0.760 (CI: 0.651–0.868); (E) NT5E: 0.889 (CI: 0.817–0.949); (F) TALDO1:0.909 (CI: 0.852–0.957). G: ROC curve of the combined model based on the training set, combining multiple biomarkers mentioned above for analysis. The AUC value of the model was 0.986 (CI: 0.972–1.000), indicating that it had a high diagnostic efficiency in the training set. H: ROC curve of the combined model based on the validation set, and the AUC value of the model was 0.992 (CI: 0.971–1.000).)

ROC curve analysis showed that a single key glycolytic gene showed a good ability to distinguish between the control group and the PAH group, and the area under the curve (AUC) and its 95% confidence interval (CI) were as follows: ACSS2: 0.876 (CI: 0.801–0.935) (Figure 7A); ALAS2: 0.850 (CI: 0.777–0.915) (Figure 7B); ALDH3A1: 0.853 (CI: 0.775–0.922) (Figure 7C); AOC3: 0.760 (CI: 0.651–0.868) (Figure 7D); NT5E: 0.889 (CI: 0.817–0.949) (Figure 7E); TALDO1: 0.909 (CI: 0.852–0.957) (Figure 7F). Among them, NT5E and TALDO1 had higher AUC values, 0.889 and 0.909, respectively. The GSE113439 and GSE117261 datasets were used as the training sets, and the GSE53408 dataset was used as the validation set. The combined model ROC curve based on the training set was combined with the above multiple biomarkers for analysis. The AUC of the model was 0.986 (CI = 0.972–1.000), indicating extremely high diagnostic performance in the training cohort (Figure 7G). Based on the ROC curve of the combined model based on the validation set, the AUC of the model was 0.992 (CI = 0.971–1.000) (Figure 7H).

**FIGURE 8:**
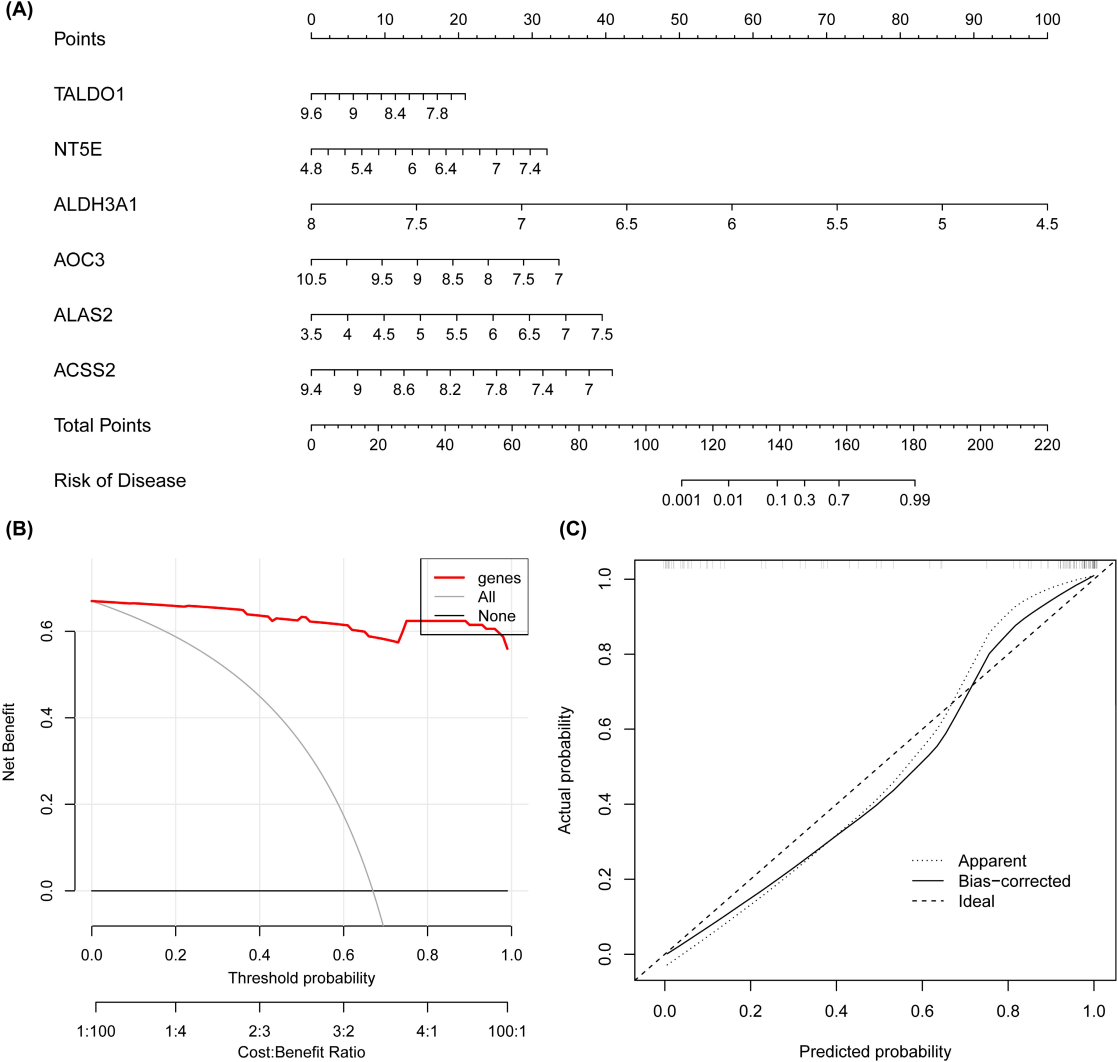
Construction of the PAH risk model. (A) Standardized nomo plot of biomarkers for predicting PAH. The integral line corresponding to each biomarker (TALDO1, NT5E, ALDH3A1, AOC3, ALAS2, ACSS2) indicates its contribution to disease risk prediction and their respective weights in the prediction model. The ‘Total Points’ row at the bottom summarizes the scores of all markers, reflecting the overall risk of the disease. The ‘Risk of Disease’ bar on the far right represents the probability of disease risk derived by the total score. (B) Decision curve analysis (DCA), showing the clinical benefits of using biomarkers for PAH risk prediction (red line) versus using no tests (grey line) or assuming everyone has the disease (black line). The threshold probability—the threshold at which a physician or patient would choose to intervene—is on the horizontal axis, and the standardized net benefit, which accounts for the benefit of a correct prediction versus the cost of an incorrect prediction, is on the vertical axis. (C) Calibration curve, showing how the predicted probability of the model corresponds to the actual probability of occurrence. The closer the curve is to the ‘ideal’ line of the grey dashed line, the more accurate the model's predictions will be. The ‘obvious’ curve (dotted line) in the figure shows the calibration of the original prediction, while the ‘bias corrected’ curve (solid line) shows the corrected prediction, reflecting the model's performance on independent data sets.

**FIGURE 9:**
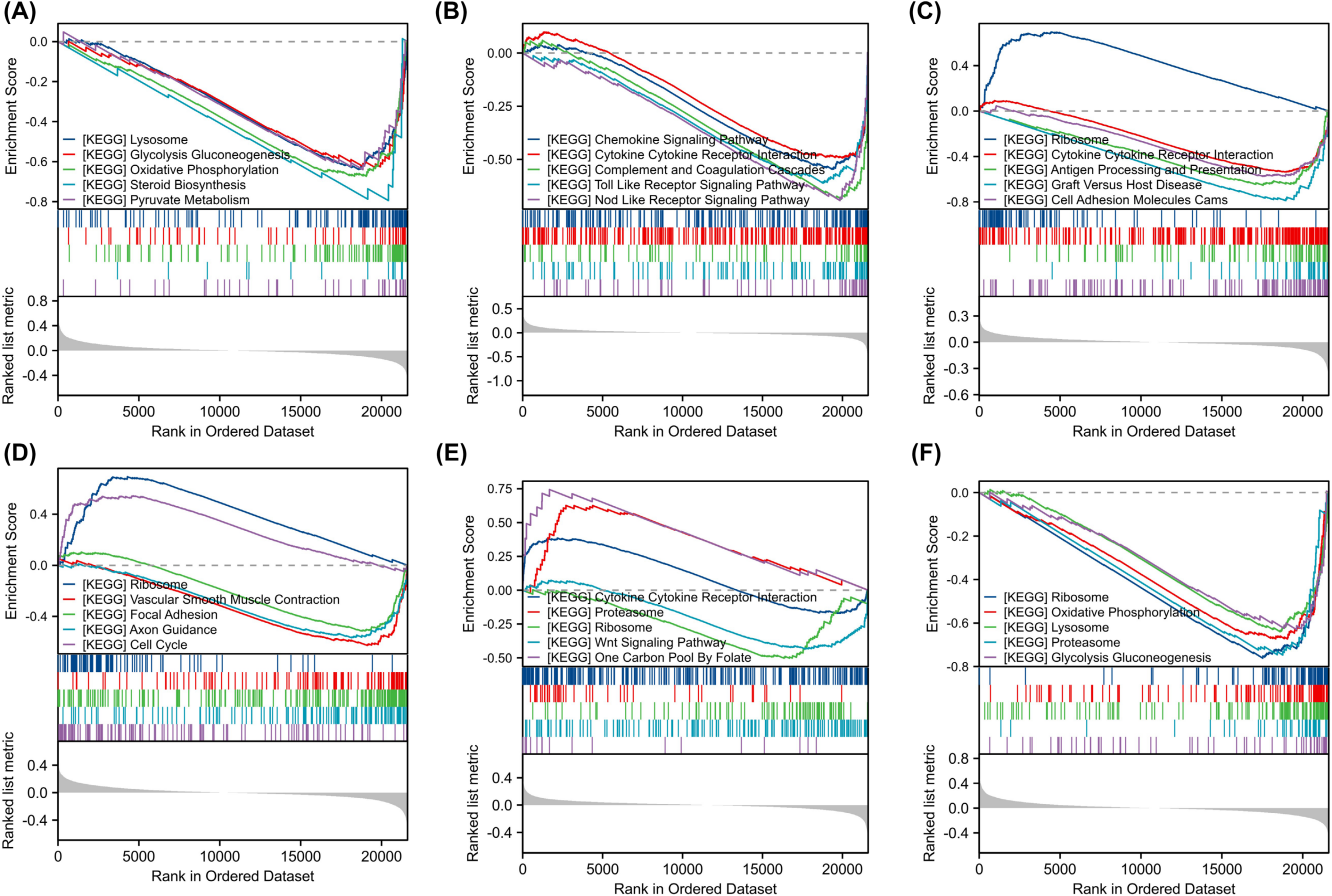
GSEA pathway enrichment analysis of high and low expression groups of biomarkers for machine learning‐assisted screening. (A) GSEA pathway enrichment analysis associated with ACSS2 markers, highlighting pathways related to lysosomes, gluconeogenesis, oxidative phosphorylation, diketose phosphate pathway, and pyruvate metabolism. (B) GSEA pathway enrichment analysis associated with ALAS2 marker, highlighting enrichment of chemokine signalling pathways, cytokine receptor interaction, complement and coagulation pathways, cell adhesion molecules and nucleotide‐binding oligomerization domain‐like receptor signalling pathways. (C) GSEA pathway enrichment analysis of ALDH3A1 markers, with particular emphasis on ribosome, cytokine‐receptor interaction, proto‐oncogene virus‐host interaction, antigen processing and presentation, and cell adhesion molecule pathways. (D) GSEA pathway enrichment analysis associated with AOC3 markers, including pathways related to ribosomes, vascular smooth muscle contraction, adhesion molecules, cell cycle and axon guidance. (E) GSEA pathway enrichment analysis associated with NT5E markers, focusing on the ribosome, proteasome, Wnt signalling pathway, adhesion molecules, and pathways driven by folate single carbon units. (F) GSEA pathway enrichment analysis of TALDO1 markers showed the enrichment status of ribosomes, oxidative phosphorylation, lysosomes, gluconeogenesis and glycolysis pathways. The upper part of the figure is the curve of the Enrichment Score (ES), indicating the degree of enrichment of the selected gene set in the entire ordered dataset. The bottom half is the ranking metric plot of the gene sets, where the colour change of the bars indicates the distribution of gene sets ranking in the data set.

The weights and disease risk prediction contributions of the six key glycolysis genes were evaluated using standardized nomograms (Figure 8A), and the clinical application value and prediction accuracy of the models were evaluated via decision curve analysis and calibration curve analysis. Decision curve analysis (DCA) revealed that risk prediction using the g‐key gene confers significant clinical benefit (Figure 8B). The calibration curve further confirmed that the bias‐corrected model had high prediction accuracy on an independent dataset (Figure 8C). GSEA revealed associations between the high‐ and low‐expression groups of six key genes and pathological pathways (Figure 9).

### 3.4 Immunological profile of PAH

**FIGURE 10:**
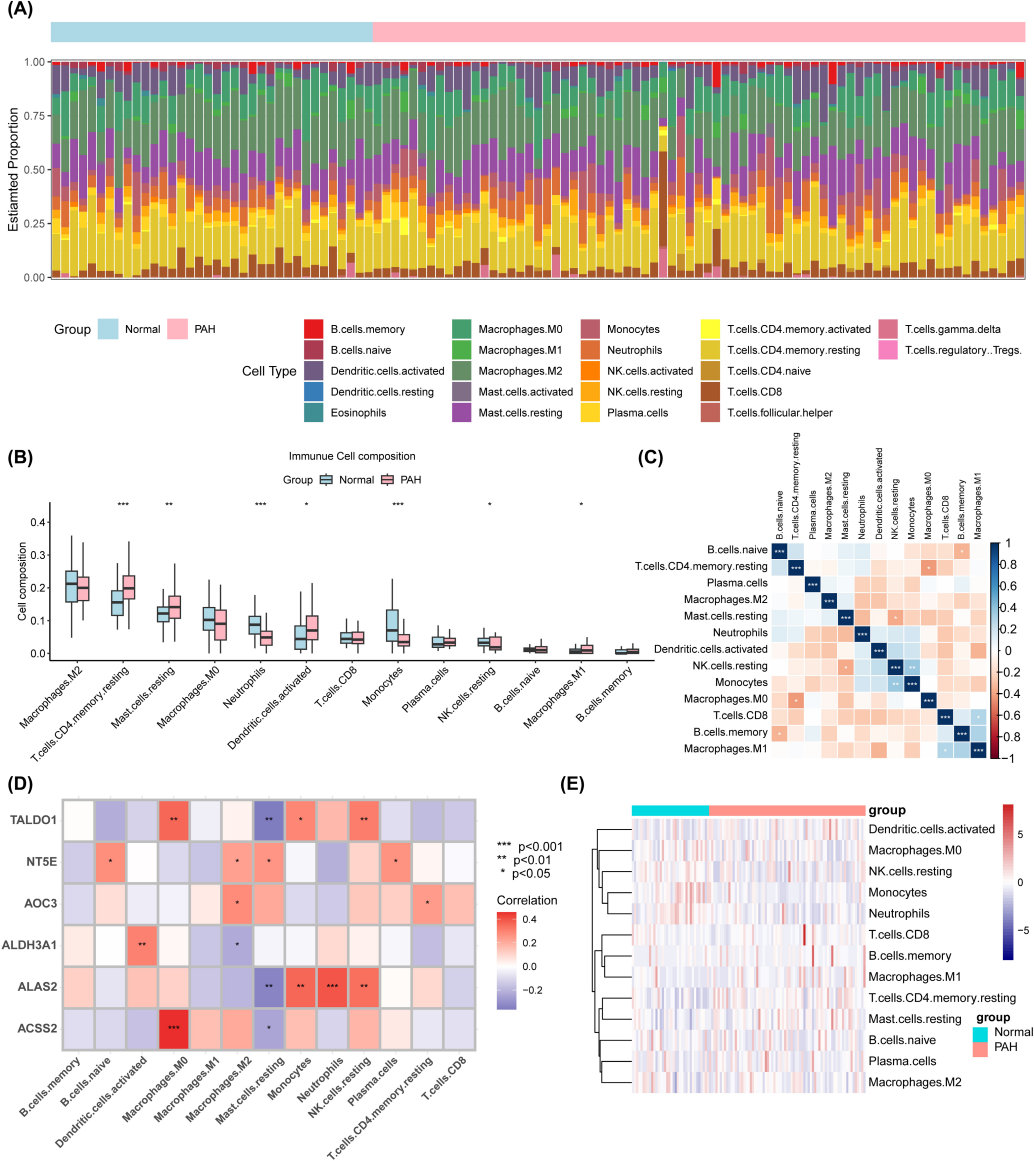
Features of immune cell infiltration in PAH. (A) The estimated proportion of immune cells is shown as a bar chart. (B) Box plot of group comparison of immune cell composition. (C) Heat map of cell type correlation matrix. This heatmap shows the correlations between different immune cell types. (D) Heat map of the correlation between biomarkers and immune cell types. This heat map shows the correlation of biomarkers ACSS2, ALAS2, ALDH3A1, AOC3, NT5E, TALDO1 with various immune cell types, where the colour indicates the strength of the correlation, red represents a positive correlation and blue represents a negative correlation. (E) Clustering heatmap of immune cell types and biomarker expression levels. This heatmap shows the pattern of expression levels of various immune cell types and biomarkers in the normal and PAH groups by cluster analysis. Colours range from blue (low expression) to red (high expression) and represent changes in expression levels. (*denotes *p* ≤ 0.05, ** denotes *p* ≤ 0.01, and *** denotes *p* ≤ 0.001).

In this study, we explored changes in the immune cell composition of PAH patients through cell‐specific gene expression analysis (Figure 10A). When analysing the composition of immune cells between the control and PAH groups, we found several statistically significant differences, including in T.cell population, memory, resting, mast.cell population, resting, dendritic.cell population, and activated macrophages. M1, The proportion of B.cell memory in the PAH group was significantly greater than that in the control group (p < 0.001). The proportion of neutrophils and monocytes in the PAH group also decreased significantly (Figure 10B). A strong positive correlation was shown between macrophages. M0 and T.cells.CD4.memory.resting (Figure 10C). A strong positive correlation was shown between B.cell memory and B.cell memory.naive, while monocyte count was negatively correlated with NK.cell resting (Figure 10C). TALDO1, NT5E, AOC3, ALDH3A1, ALAS2, and ACSS2 showed significant correlations with the abundance of different immune cell type (Figure 10D). The cluster heatmap illustrates the different distribution patterns of immune cell types between the control and PAH groups (Figure 10E).

### 3.5 Machine learning‐assisted screening of biomarkers and miRNA, TF and small molecule drug action networks

**FIGURE 11:**
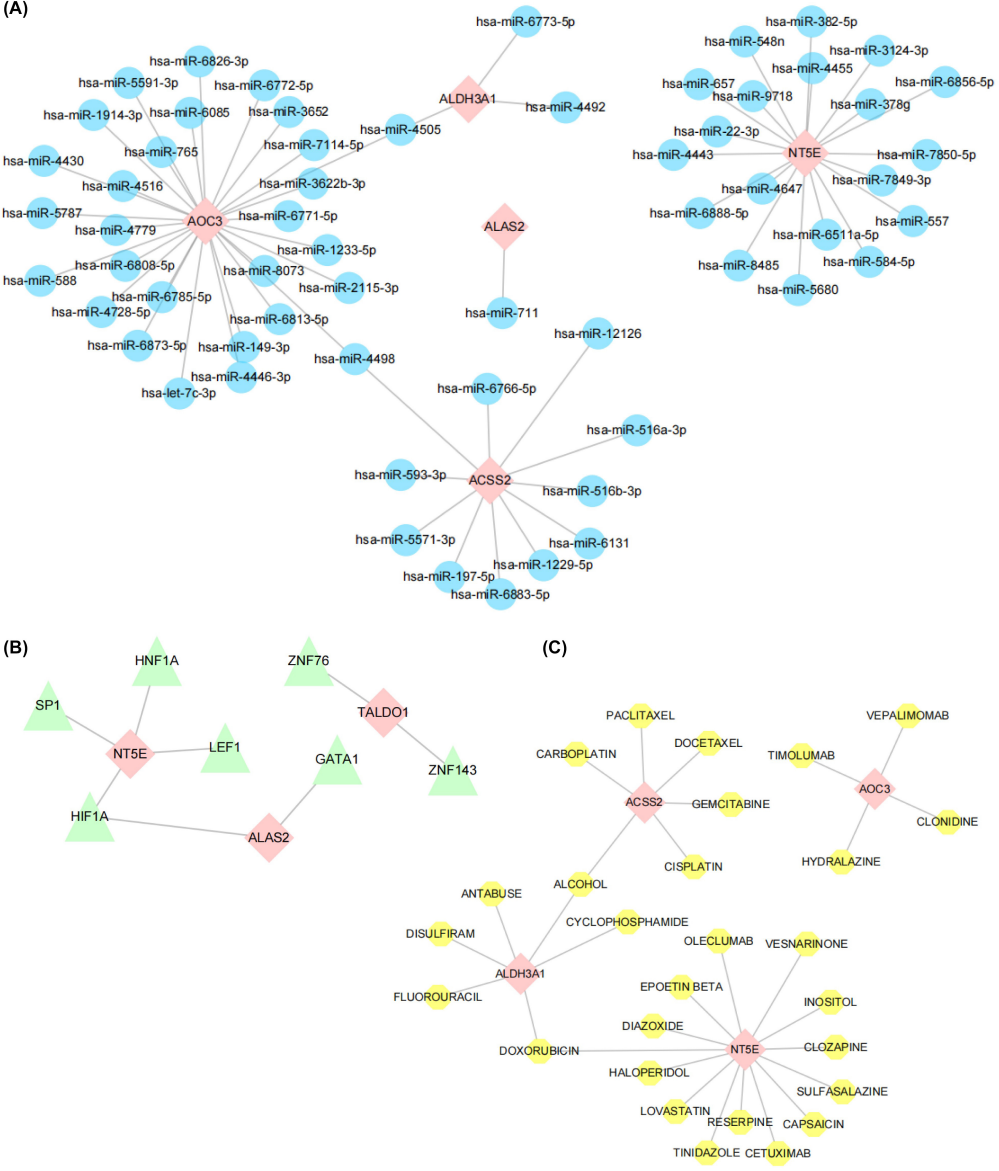
Machine learning‐assisted screening of biomarkers with miRNA, TF, and small molecule drug interaction network. (A–C) Schematic representation of the protein structures encoded by ACSS2, ALAS2, ALDH3A1, AOC3, NT5E, TALDO1. (A) biomarker and miRNA interaction network. The network diagram shows the interaction between the selected biomarkers (such as ALDH3A1, ALAS2, ACSS2, NT5E, TALDO1) and different mirnas (such as hsa‐miR‐558, hsa‐miR‐6785‐5p, hsa‐miR‐4443, etc.). Nodes in the network represent biomarkers or mirnas, and lines represent potential interactions or regulatory relationships between them. (B) biomarker‐transcription factor (TF) interaction network. The network diagram shows the interaction between biomarkers and different TFS, such as ALAS2 with HIF1A and LEF1; NT5E with SP1 and HNF1A; And TALDO1 with GATA1 and ZNF143. Each line represents a potential regulatory relationship. (C) biomarker and small molecule drug action network. This figure reveals the interaction between biomarkers and a variety of small molecule drugs, such as ALDH3A1 with DIAZOXIDE and CYCLOPHOSPHAMIDE; ACSS2 versus GEMCITABINE and CISPLATIN; AOC3 and CLOZAPINE, SULFASALAZINE. The lines represent potential biomarker sites of action for the drug.

**FIGURE 12:**
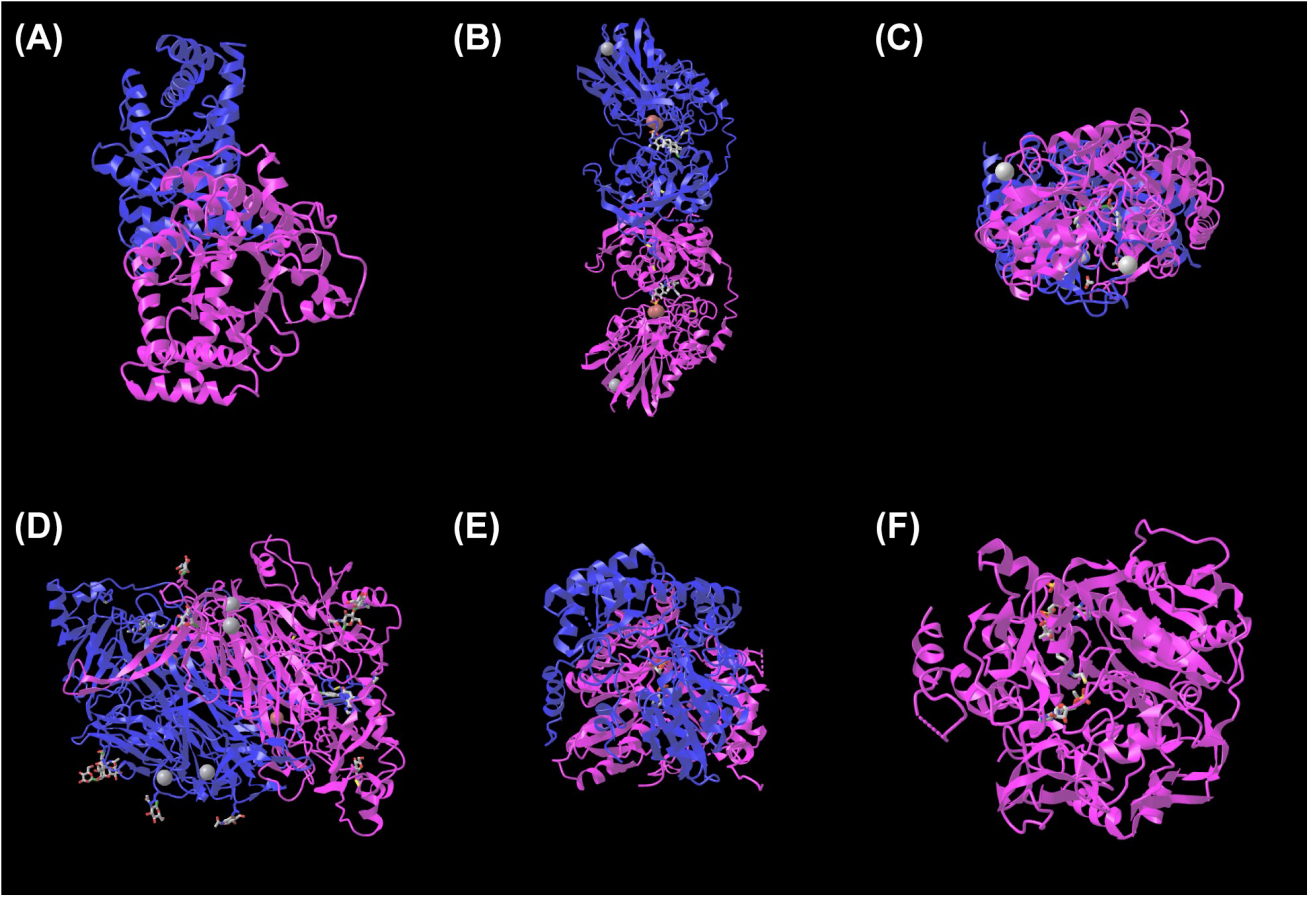
Schematic diagram of protein structures of biomarkers for machine learning‐assisted screening. (A) Three‐dimensional structure of Acetyl‐CoA Synthetase 2‐Acetyl‐coa synthetase 2. (B) Spatial configuration of 5’‐Aminolevulinate Synthase 2‐5‐aminolevulinate synthetase 2. (C) Aldehyde Dehydrogenase 3 Family, Member A1‐3D conformation of aldehyde dehydrogenase 3 family member A1. (D) Protein folding model of Amine Oxidase, Copper Containing 3‐Amine oxidase copper containing 3. (E) Protein stereoscopic structure of 5′‐nucleotidase‐5‘‐Nucleotidase. (F) 3D model of Transaldolase 1‐transketouronoate‐3 phosphate isomerase 1.

An mRNA–miRNA network of glycolysis‐related genes was constructed using a network database containing five mRNAs and 60 miRNAs (Figure 11A). The mRNA‐TF network of glycolysis‐related hub genes, which included three mRNAs and eight TFs, was constructed from the TRRUST database (Figure 11B). Potential drugs or small molecule compounds containing four mRNAs and 30 small molecule drugs containing four mRNAs and 30 small molecule drugs were predicted by the DGIdb version 3.0.2 (https://www.dgidb.org) (Figure 11C). The detailed three‐dimensional structures of several key metabolic enzymes were revealed by the MMDB (Figure 12A–F).

## 4 DISCUSSION

Pulmonary arterial hypertension (PAH) is a common lung disease, and the molecular mechanism underlying the occurrence and development of PAH has not been fully elucidated thus far. Moreover, PAH diagnosis and evaluation rely mainly on echocardiography and right heart catheterization, and there is an urgent need to discover additional potential markers for effective prognostic evaluation.^26^ With increasing attention given to the study of energy metabolism, PAH and energy metabolism are also inextricably linked, and metabolic changes and biodynamic changes are common characteristics of patients and animal models of PAH disease. For example, aerobic glycolysis has been observed in pulmonary artery endothelial cells (PAECs) and smooth muscle cells (PASMCs) in patients with idiopathic pulmonary hypertension (IPAH) and in rodent PH models,^27^ and an abnormal increase in the glycolytic pathway^28^ has also occurred during pulmonary vascular remodelling.

To explore the relationship between glycolysis and the occurrence and development of PAH, DEGs were identified via bioinformatics mining of the GSE113439, GSE117261, and GSE53408 datasets in the GEO database. We also found that the significant adjustment of multiple biological metabolic pathways associated with PAH was achieved through GO functional enrichment and KEGG pathway enrichment. The results showed that glycolysis genes involved in the occurrence and development of PAH play synergistic roles in multiple pathways.

In this study, Lasso regression and SVM‐RFE machine learning technology were combined and applied for the first time to screen six glycolytic DEGs, namely ACSS2, ALAS2, AOC3, ALDH3A1, TALDO1, and NT5E, were screened. According to the ROC curve analysis, individual biomarkers showed good ability to distinguish between normal controls and PAH patients, with NT5E and TALDO1 having higher AUC values. TALDO1 plays a crucial role in the pentose phosphate pathway (PPP) during glucose oxidation. The expression of TALDO in the PPP of the oxidation phase contributes to the generation of NADPH and is very important for maintaining the balance of metabolites.^29^ The oxidation phase‐transfer aldol enzyme acts as a catalyst in the group‐transfer reaction and generates a large amount of 6‐phosphoric acid fructose through glycolytic pathway oxidation decomposition, so the oxidation of the PPP in these stages is a branch of glycolysis. The most important function of this pathway is not to generate an energy supply but rather to generate ribose 5‐phosphate (R5P), which provides important raw materials for the synthesis of nucleotides, adenosine triphosphate (ATP), acetyl‐CoA (CoA), and nicotinamide adenine dinucleotide (NAD+) and generates a large amount of reduced equivalent NADPH. NADPH is used in the body's biosynthetic reactions and maintains glutathione (GSH) in a reduced state to protect against oxidative stress. Therefore, it participates in various metabolic reactions in the body as a hydrogen donor. The protective effect of antioxidant stress on the body is closely related to PAH.^30^ Therefore, it is theoretically speculated that the downregulation of TALDO1 may lead to the occurrence and development of PAH. LinWencha et al. reported that the expression of TALDO1 was inhibited in the IPAH group compared with the normal group,^31^ which is similar to our results.

NT5E is currently a potential target for tumour therapy, and its encoded CD73 protein is a key enzyme involved in AMP hydrolysis to adenosine.^32^ Adenosine is a retaliatory metabolite that is expressed in conditions of injury or stress. Adenosine receptors can also promote the differentiation of lung fibroblasts into myofibroblasts, which is typical of fibrotic events. This last function suggests the potential involvement of adenosine in fibrotic lung disease processes, which are characterized by different degrees of inflammation and fibrosis. In addition, adenosine may also be involved in the process of fibrotic lung diseases characterized by varying degrees of inflammation and fibrosis.^33^ In addition, studies have also confirmed that ALDH1A3 in the ALDH3A1 family participates in glycolysis in PASMCs to regulate the proliferation of smooth muscle cells.^34^ We believe that these six key genes have great value and significance as diagnostic and therapeutic markers for PAH.

On this basis, we used standardized nomograms to evaluate the weights and disease risk prediction contributions of each key gene, and the decision curve analysis showed that the use of key genes for risk prediction can provide significant clinical benefits. The calibration curve further confirmed that the bias‐corrected model had high prediction accuracy on an independent dataset. This is a landmark improvement in the accuracy of PAH risk prediction, reflecting our innovative attempt to solve complex biomedical problems using modern computational methods. Therefore, the pathogenesis of PAH can be clarified by exploring the function of these genes in the future.

We also performed GSEA to explore the associations between different biomarker expression groups and pathological pathways. The ACSS2 gene was significantly enriched in key metabolic pathways, such as lysosomes, gluconeogenesis, oxidative phosphorylation, the diketose phosphate pathway, and pyruvate metabolism, suggesting that these metabolic processes may play important roles in the occurrence and development of PAH. Analysis of the ALAS2 gene highlighted the enrichment of immune‐related pathways, such as chemokine signalling pathways and cell adhesion molecules, reflecting the potential influence of the immune system in disease. The relevant enrichment results for ALDH3A1 highlighted ribosomal and cytokine receptor interactions, indicating the importance of protein synthesis and signalling in disease. Analysis of AOC3 revealed significant enrichment of ribosome, vascular smooth muscle contraction, and cell cycle pathway terms, which may be associated with key pathological features of PAH, such as vascular remodelling. NT5E analysis focused on the ribosome, proteasome, and Wnt signalling pathways, which underlie cell growth, differentiation, and signal transduction. The TALDO1 results revealed enrichment of energy metabolism pathways, such as glycolysis and oxidative phosphorylation pathways, suggesting that abnormal energy metabolism may play a central role in the pathogenesis of PAH.

An increasing number of studies indicate that immune cell infiltration is crucial for the occurrence and development of PAH.^35^ To further investigate the function of immune cells in PAH, we analysed the composition of immune cells in patients with PAH through CIBERSORTx technology, which not only enhanced our understanding of the immune regulation mechanism of PAH, but also showed an innovative research method to explore the immunological characteristics of the disease using advanced bioinformatics tools. The results showed that the proportions of resting CD4+ memory cells, resting mast cells, activated dendritic cells, M1 macrophages, and memory B cells were significantly greater in the PAH group than in the normal group (*p* < 0.001). The proportion of neutrophils and monocytes in the PAH group also decreased significantly. Shengxin Tang et al. also studied the functional characteristics of immune cells in PAH patients, and the results showed that NK cell activation; monocyte and T‐cell CD4 memory activation; and mast cell infiltration were increased and that T‐cell CD4 naive infiltration was decreased in the peripheral blood of PAH patients compared with those in the control group.^36^ These results are similar to our findings; both of these findings reveal significant differences in the number of specific types of immune cells in PAH patients compared to that in healthy individuals, which may reflect changes in immunomodulation during the pathological process of PAH. In addition, correlation analysis revealed complex interactions between different immune cell types, with a strong positive correlation between M0 macrophages and resting CD4 memory T cells. There was a strong positive correlation between B‐cell memory and naive B cells, while monocyte count was negatively correlated with resting NK cells. Further analysis revealed that the key genes TALDO1, NT5E, AOC3, ALDH3A1, ALAS2 and ACSS2 were significantly correlated with the abundance of different immune cell types. For example, the expression of ALDH3A1 was positively correlated with the abundance of activated Dendritic cells, while the expression of ACSS2 was positively correlated with the number of macrophages. M0 cells. In addition, the cluster heatmap shows the different distribution patterns of immune cell types between the normal and PAH groups, highlighting significant changes in the immune cell profile in the state of PAH. Therefore, we speculate that TALDO1, NT5E, ALDH3A1, AOC3, ALAS2, and ACSS2 are associated with the occurrence and progression of PAH by regulating multiple immune cells. However, additional research is needed to determine the complex interactions between genes and immune cells.

In this study, we also revealed the detailed three‐dimensional structures of several key metabolic enzymes through the use of MMDB. Using the resources of the Molecular Modelling Database (MMDB), we demonstrated the precise three‐dimensional structure of the core metabolic enzymes in this study. The three‐dimensional structure of acetyl‐CoA synthetase 2, encoded by ACSS2, reveals a typical cleavage synthase folding pattern with a deep substrate binding pocket, suggesting that this protein may have a high degree of substrate specificity. The ALAS2‐encoded 5‐aminolevulinate synthetase 2 model contains a key iron–sulfur cluster binding site, which is essential for its function in the process of heme synthesis. The three‐dimensional conformation of the ALDH3A1 gene encodes aldehyde dehydrogenase 3 family member A1, which has a broad NAD(P) + binding domain, highlighting its potential as a multifunctional enzyme in metabolic pathways. The structure of the amine oxidase encoded by AOC33 contains a specific copper binding site, which is an integral part of its catalytic oxidation of amine molecules. The three‐dimensional structure of the 5′‐nucleotidase encoded by NT5E reveals a key phosphoric hydrolytic active site that plays an important role in regulating extracellular nucleotide levels. Finally, the model of transketouronic acid‐3 phosphoisomerase 1 encoded by TALDO1 highlights its key catalytic role in the PPP, which is needed for the synthesis of intracellular nucleic acids and fatty acids. The detailed presentation of these structures not only further confirms their biological functions but also provides a solid foundation for future drug design and functional studies of these targets. The discovery of these structures provides important information for further drug development and functional studies of these enzymes.

This study has certain limitations: first, this study did not conduct an integrated analysis of PAH related tissues; secondly, the sample size is small, and the three geo datasets selected are microarray datasets, while single microarray analysis may lead to high false positive rate and one‐sided results; third, due to the heterogeneity of PAH and the lack of clinical data, we could not evaluate the association between risk indicators and patient stratification based on PAH severity; fourth, not all patients with PAH have consistent symptoms and biomarker performance. These changes may only occur in a subgroup of PAH patients with specific clinical and pathophysiological characteristics. Therefore, in the future, we need to further increase the sample size for external validation to determine the diagnostic accuracy of key genes related to PAH, and also need further experimental evidence, such as real‐time PCR, Western blot, and immunohistochemical analysis, to fully clarify the role of key genes and the potential mechanism of PAH.

In this study, we first identified and comprehensively analysed the DEGs associated with glycolysis in PAH patients via the use of the GEO database and screened six key glycolysis genes for the diagnosis of PAH via machine learning, and the results showed satisfactory predictive performance.

## AUTHOR CONTRIBUTIONS

**Yu‐xuan Lou:** Conceptualization (equal); data curation (equal); writing – original draft (lead). **Er‐dan Shi:** Data curation (equal); funding acquisition (equal). **Rong Yang:** Conceptualization (equal); software (equal); writing – original draft (supporting). **Yang Yang:** Conceptualization (equal); data curation (equal); methodology (equal); writing – review and editing (lead).

## FUNDING INFORMATION

This work was supported by the Science and Technology Project of Jiangsu Province Hospital of Chinese Medicine (No. Y22060).

## CONFLICT OF INTEREST STATEMENT

The authors have no conflicts of interest to declare.

## Data Availability

The data generated or analysed during this study are included in this published article, original data are available from the Gene Expression Omnibus database.

## References

[1] McGoon MD , Benza RL , Escribano‐Subias P , et al. Pulmonary arterial hypertension: epidemiology and registries. J Am Coll Cardiol. 2013;62(25 Suppl):D51‐D59. doi:10.1016/j.jacc.2013.10.023 24355642

[2] Sun T , Yuan W , Wei Y , Liao D , Tuo Q . The regulatory role and mechanism of energy metabolism in vascular diseases. Front Biosci. 2024;29(1):26. doi:10.31083/j.fbl2901026 38287818

[3] Chen S , Zou Y , Song C , et al. The role of glycolytic metabolic pathways in cardiovascular disease and potential therapeutic approaches. Basic Res Cardiol. 2023;118(1):48. doi:10.1007/s00395-023-01018-w 37938421 PMC10632287

[4] Kovacs L , Cao Y , Han W , et al. PFKFB3 in smooth muscle promotes vascular remodeling in pulmonary arterial hypertension. Am J Respir Crit Care Med. 2019;200(5):617‐627. doi:10.1164/rccm.201812-2290OC 30817168 PMC6727156

[5] Liang S , Yegambaram M , Wang T , Wang J , Black SM , Tang H . Mitochondrial metabolism, redox, and calcium homeostasis in pulmonary arterial hypertension. Biomedicines. 2022;10(2):341. doi:10.3390/biomedicines10020341 35203550 PMC8961787

[6] Cao Y , Zhang X , Wang L , et al. PFKFB3‐mediated endothelial glycolysis promotes pulmonary hypertension. Proc Natl Acad Sci USA. 2019;116(27):13394‐13403. doi:10.1073/pnas.1821401116 31213542 PMC6613097

[7] Wang J , Liu C , Huang SS , et al. Functions and novel regulatory mechanisms of key glycolytic enzymes in pulmonary arterial hypertension. Eur J Pharmacol. 2024;970:176492. doi:10.1016/j.ejphar.2024.176492 38503401

[8] Mainali S , Darsie ME , Smetana KS . Machine learning in action: stroke diagnosis and outcome prediction. Front Neurol. 2021;12:734345. doi:10.3389/fneur.2021.734345 34938254 PMC8685212

[9] Liao Y , Yang Y , Zhou G , et al. Anoikis and SPP1 in idiopathic pulmonary fibrosis: integrating bioinformatics, cell, and animal studies to explore prognostic biomarkers and PI3K/AKT signaling regulation. Expert Rev Clin Immunol. 2024;6:1‐15. doi:10.1080/1744666X.2024.2315218 38318669

[10] Liao Y , Wang R , Wen F . Diagnostic and prognostic value of secreted phosphoprotein 1 for idiopathic pulmonary fibrosis: a systematic review and meta‐analysis. Biomarkers. 2023;28(1):87‐96. doi:10.1080/1354750X.2022.2148744 36377416

[11] Mura M , Cecchini MJ , Joseph M , Granton JT . Osteopontin lung gene expression is a marker of disease severity in pulmonary arterial hypertension. Respirology. 2019;24(11):1104‐1110. doi:10.1111/resp.13557 30963672

[12] Zhao YD , Yun H , Peng J , et al. De novo synthesize of bile acids in pulmonary arterial hypertension lung. Metabolomics. 2014;10(6):1169‐1175. doi:10.1007/s11306-014-0653-y 25374487 PMC4213391

[13] Stearman RS , Bui QM , Speyer G , et al. Systems analysis of the human pulmonary arterial hypertension lung transcriptome. Am J Respir Cell Mol Biol. 2019;60(6):637‐649. doi:10.1165/rcmb.2018-0368OC 30562042 PMC6543748

[14] Leek JT , Johnson WE , Parker HS , Jaffe AE , Storey JD . The sva package for removing batch effects and other unwanted variation in high‐throughput experiments. Bioinformatics. 2012;28(6):882‐883. doi:10.1093/bioinformatics/bts034 22257669 PMC3307112

[15] Ritchie ME , Phipson B , Wu D , et al. Limma powers differential expression analyses for RNA‐sequencing and microarray studies. Nucleic Acids Res. 2015;43(7):e47. doi:10.1093/nar/gkv007 25605792 PMC4402510

[16] Subramanian A , Tamayo P , Mootha VK , et al. Gene set enrichment analysis: a knowledge‐based approach for interpreting genome‐wide expression profiles. Proc Natl Acad Sci USA. 2005;102(43):15545‐15550. doi:10.1073/pnas.0506580102 16199517 PMC1239896

[17] Yu G , Wang LG , Han Y , He QY . ClusterProfiler: an R package for comparing biological themes among gene clusters. OMICS. 2012;16(5):284‐287. doi:10.1089/omi.2011.0118 22455463 PMC3339379

[18] Liberzon A , Birger C , Thorvaldsdottir H , Ghandi M , Mesirov JP , Tamayo P . The molecular signatures database (MSigDB) hallmark gene set collection. Cell Syst. 2015;1(6):417‐425. doi:10.1016/j.cels.2015.12.004 26771021 PMC4707969

[19] Friedman J , Hastie T , Tibshirani R . Regularization paths for generalized linear models via coordinate descent. J Stat Softw. 2010;33(1):1‐22. 20808728 PMC2929880

[20] Duan KB , Rajapakse JC , Wang H , Azuaje F . Multiple SVM‐RFE for gene selection in cancer classification with expression data. IEEE Trans Nanobioscience. 2005;4(3):228‐234. doi:10.1109/tnb.2005.853657 16220686

[21] Vickers AJ , Elkin EB . Decision curve analysis: a novel method for evaluating prediction models. Med Decis Mak. 2006;26(6):565‐574. doi:10.1177/0272989X06295361 PMC2577036 17099194

[22] Newman AM , Steen CB , Liu CL , et al. Determining cell type abundance and expression from bulk tissues with digital cytometry. Nat Biotechnol. 2019;37(7):773‐782. doi:10.1038/s41587-019-0114-2 31061481 PMC6610714

[23] Sticht C , De La Torre C , Parveen A , Gretz N . MiRWalk: an online resource for prediction of microRNA binding sites. PLoS One. 2018;13(10):e206239. doi:10.1371/journal.pone.0206239 PMC6193719 30335862

[24] Han H , Cho JW , Lee S , et al. TRRUST v2: an expanded reference database of human and mouse transcriptional regulatory interactions. Nucleic Acids Res. 2018;46(D1):D380‐D386. doi:10.1093/nar/gkx1013 29087512 PMC5753191

[25] Madej T , Lanczycki CJ , Zhang D , et al. MMDB and VAST+: tracking structural similarities between macromolecular complexes. Nucleic Acids Res. 2014;42(Database issue):D297‐D303. doi:10.1093/nar/gkt1208 24319143 PMC3965051

[26] Humbert M , Kovacs G , Hoeper MM , et al. 2022 ESC/ERS guidelines for the diagnosis and treatment of pulmonary hypertension. Eur Heart J. 2022;43(38):3618‐3731. doi:10.1093/eurheartj/ehac237 36017548

[27] Hernandez‐Saavedra D , Sanders L , Freeman S , et al. Stable isotope metabolomics of pulmonary artery smooth muscle and endothelial cells in pulmonary hypertension and with TGF‐beta treatment. Sci Rep. 2020;10(1):413. doi:10.1038/s41598-019-57200-5 31942023 PMC6962446

[28] Rehman J , Archer SL . A proposed mitochondrial‐metabolic mechanism for initiation and maintenance of pulmonary arterial hypertension in fawn‐hooded rats: the Warburg model of pulmonary arterial hypertension. Adv Exp Med Biol. 2010;661:171‐185. doi:10.1007/978-1-60761-500-2_11 20204730

[29] Perl A , Hanczko R , Telarico T , Oaks Z , Landas S . Oxidative stress, inflammation and carcinogenesis are controlled through the pentose phosphate pathway by transaldolase. Trends Mol Med. 2011;17(7):395‐403. doi:10.1016/j.molmed.2011.01.014 21376665 PMC3116035

[30] Xu D , Hu YH , Gou X , et al. Oxidative stress and antioxidative therapy in pulmonary arterial hypertension. Molecules. 2022;27(12):3724. doi:10.3390/molecules27123724 35744848 PMC9229274

[31] Lin W , Tang Y , Zhang M , et al. Integrated bioinformatic analysis reveals TXNRD1 as a novel biomarker and potential therapeutic target in idiopathic pulmonary arterial hypertension. Front Med (Lausanne). 2022;9:894584. doi:10.3389/fmed.2022.894584 35646965 PMC9133447

[32] Ghalamfarsa G , Kazemi MH , Raoofi MS , et al. CD73 as a potential opportunity for cancer immunotherapy. Expert Opin Ther Targets. 2019;23(2):127‐142. doi:10.1080/14728222.2019.1559829 30556751

[33] Della LV , Cabiati M , Rocchiccioli S , Del RS , Morales MA . The role of the adenosinergic system in lung fibrosis. Pharmacol Res. 2013;76:182‐189. doi:10.1016/j.phrs.2013.08.004 23994158

[34] Xie X , Urabe G , Marcho L , Stratton M , Guo LW , Kent CK . ALDH1A3 regulations of matricellular proteins promote vascular smooth muscle cell proliferation. iScience. 2019;19:872‐882. doi:10.1016/j.isci.2019.08.044 31513972 PMC6739626

[35] Wang RR , Yuan TY , Wang JM , et al. Immunity and inflammation in pulmonary arterial hypertension: from pathophysiology mechanisms to treatment perspective. Pharmacol Res. 2022;180:106238. doi:10.1016/j.phrs.2022.106238 35504356

[36] Tang S , Liu Y , Liu B . Integrated bioinformatics analysis reveals marker genes and immune infiltration for pulmonary arterial hypertension. Sci Rep. 2022;12(1):10154. doi:10.1038/s41598-022-14307-6 35710932 PMC9203517

